# Study on anxiety, depression, and subjective wellbeing of patients with bladder cancer in their different chemotherapy stages

**DOI:** 10.3389/fpsyg.2023.1226712

**Published:** 2023-09-13

**Authors:** Jie Yang, Yingchun Tan, Chunlin Yao

**Affiliations:** ^1^School of Nursing and Health, Caofeidian College of Technology, Tangshan, China; ^2^Department of School Clinic, Tianjin Chengjian University, Tianjin, China; ^3^School of Foreign Languages, Tianjin Chengjian University, Tianjin, China

**Keywords:** anxiety, bladder cancer, depression, perfusion chemotherapy, subjective wellbeing

## Abstract

**Aims and objectives:**

This study aims to explore the changes in anxiety, depression, and subjective wellbeing in patients with bladder perfusion during different stages of their chemotherapy, and analyze the correlation among their anxiety, depression, and subjective wellbeing.

**Methods:**

A total of 174 patients with bladder cancer who received bladder perfusion chemotherapy were selected. The questionnaire survey was conducted with SAS, SDS, and GWB. The patients were surveyed and the data were analyzed.

**Results:**

The results of patients' anxiety, depression, and subjective wellbeing show a dynamic change trend. The change is most obvious after 1 month of chemotherapy and tends to be flat after 3 months of chemotherapy. The scores of anxiety, depression, and subjective wellbeing in patients with bladder perfusion at different stages are statistically significant (*P* < 0.05). There is a negative correlation among anxiety, depression, and subjective wellbeing in patients (*r* = −0.605, 0.601).

**Conclusion:**

Patients' emotions change obviously in the first 3 months of their chemotherapy. Clinical workers can take active intervention measures in this period, guide patients to relieve their anxiety and depression, increase their subjective wellbeing level, and improve their quality of life, which is helpful to ensure the successful completion of chemotherapy.

## 1. Introduction

Bladder cancer is the most common malignant tumor of the urinary system. Approximately 70–80% of patients have non-muscular invasive bladder cancer (NMIBC) (Bhanvadia, 2018), which needs to be treated by bladder tumor resection combined with bladder perfusion chemotherapy. Bladder infusion chemotherapy lasts for a long time, which results in the consequence that patients are prone to negative emotions such as anxiety and depression. Some patients will lose confidence in their treatment and delay or even give up chemotherapy, which affects their quality of life (Tang et al., 2019; Vartolomei et al., 2020). Negative emotions affect the body's neuroendocrine and immune regulatory functions, leading to disorders of the immune defense system, and increasing the risk of tumor cell growth and spread (Liu L. et al., 2020). As part of positive psychology, subjective wellbeing has a certain positive effect on cancer patients' quality of life (Edelstein et al., 2016). Previous studies on patients' negative emotions and subjective wellbeing are mostly limited to the investigation of the influencing factors (Li and Wang, 2016; Smith et al., 2018; Xu et al., 2019). Without longitudinal studies, it is impossible to understand the specific changes in patients during their chemotherapy and rehabilitation. This study will analyze the changes in anxiety, depression, and subjective wellbeing in NMIBC patients during their bladder infusion and try to find the correlation among them. The research results can help chemotherapy patients relieve their anxiety and depression, improve their subjective wellbeing and quality of life, ensure the smooth completion of chemotherapy, and provide theoretical support for clinical psychology interventions.

## 2. Methods

This is an experimental study approved by the Committee of Ethics and Integrity in Research with Humans at Tianjin Chengjian University, China. In addition, written informed consent was obtained from the participant for the publication of this report (including all data and images).

### 2.1. Participants and sample

The study chooses samples from Tangshan Gongren Hospital and Tangshan People's Hospital. Both the hospitals are 3A level hospitals in Tangshan City, the uppermost level hospital in China. Patients with bladder cancer who underwent bladder infusion chemotherapy between the periods of August 2020 and May 2021 are the potential research samples in this study. The inclusion criteria for selecting research participants in this study are as follows: (1) patients with NMIBC who met the clinical diagnosis of bladder cancer (DeGeorge et al., 2017); and (2) patients with bladder tumor resection combined with bladder perfusion chemotherapy. The exclusion criteria for selecting research participants in the study are as follows: (1) patients with other cancers; and (2) patients with cognitive impairment and unable to cooperate.

The study adopts a random sampling method. The 5th and 15th potential research samples who go to Tangshan Gongren Hospital on Tuesday or Tangshan People's Hospital on Friday from August 2020 to May 2021 are selected as the research samples. If the number of potential research samples on 1 day is more than 25, the 25th sample is chosen as the research sample as well. During the research period, the study selected 180 potential samples in total and sent out 180 questionnaires. Finally, it received 174 valid questionnaires with a recovered effective rate of 96.7%. The 174 patients who responded to the investigation validly are the research samples.

### 2.2. Instruments

The data are collected with a questionnaire, which contains four sections. The first section is about demographic information, such as age, gender, education level, perfusion drugs, recurrence, and time of chemotherapy.

The second section is the Self-Rating Anxiety Scale (SAS), consisting of 20 items that measure the levels of anxiety symptoms (Yan et al., 2019). The scale stipulates that a score <50 represents no anxiety symptoms, from 50 to 59 mild anxiety, from 60 to 69 moderate anxiety, and more than 70 severe anxiety. The Cronbach's α of this scale is 0.777, which means the scale is highly reliable.

The third section is the Self-Rating Depression Scale (SDS), consisting of 20 items that measure the levels of the participant's depressive symptoms (Huang et al., 2019). The scale can be divided into four levels: no depression (with a score <53), mild depression (with a score between 54 and 63), moderate depression (with a score between 64 and 73), and major depression (with a score more than 73). The Cronbach's α of this scale is 0.782, which indicates the scale is highly reliable.

The last section is the General Wellbeing Schedule (GWB), which contains six dimensions (Lau et al., 2016). The higher the score is, the happier the investigatee feels. The Cronbach's α of this scale is 0.850, which shows the scale is highly reliable.

Three experts (all of them are Full Professors of Psychology) have examined the validity of the questionnaire. They confirm that the questionnaire has high validity in evaluating patients' anxiety, depression, and wellbeing.

At the same time, Excel software is used for data entry, and the data are analyzed with the help of the software SPSS25.0. The GraphPad Prism 9.0 plotting software is used to draw statistical graphs.

### 2.3. Ethical considerations

A specially trained researcher issues the questionnaire in the cystoscope room of the urology department and explains to the patients the purpose of the survey and some matters that need to be paid attention to during the investigation. The patients are instructed to fill in the questionnaire.

### 2.4. Data analysis

Technical data are expressed by frequency and percentage, and measurement data are expressed by mean value ± standard deviation. ANOVA and LSD-t multiple comparisons are used for further analysis of the data.

## 3. Results

Among the 174 patients, 124 (71.3%) are male patients and 50 (28.7%) female patients; 53 (30.5%) are relapsed patients and 121 (69.5%) first-time patients; and 44 (25.3%) are non-chemotherapy patients, 38 (21.8%) with 1 month of chemotherapy, 49 (28.2%) with 3 months of chemotherapy, 21 (12.1%) with 6 months of chemotherapy, and 22 (12.6%) with 12 months of chemotherapy.

### 3.1. Comparison of patients' anxiety, depression, and subjective wellbeing

The average score of patients' anxiety before chemotherapy is 53.09 ± 9.370, at a mild anxiety level. After 12 months of chemotherapy, the average score drops to 45.05 ± 7.937, which shows these patients are in an anxiety-free state. The average score of patients' depression before chemotherapy is 56.34 ± 11.759, at a mild depression level. After 12 months of chemotherapy, the average score drops to 45.64 ± 7.865, which indicates the patients are in a depression-free state. The average score of the patient's subjective wellbeing is 73.59 ± 8.171 before chemotherapy. After 12 months of chemotherapy, it rises to 80.14 ± 5.557. Table 1 reveals the development of the patient's anxiety levels, depression levels, and subjective wellbeing levels before chemotherapy and 12 months of chemotherapy.

**Table 1:** Patient's anxiety, depression, and subjective wellbeing before chemotherapy and after 12 months of chemotherapy (*N* = 174).

**Variable**	**Measures**	**M ±SD**	***F***	***P***
Anxiety	Baseline	53.09 ± 9.370	11.025	0.000
	12 months	45.05 ± 7.937		
Depression	Baseline	56.89 ± 10.262	13.561	0.000
	12 months	47.95 ± 5.996		
Subjective wellbeing	Baseline	72.84 ± 9.185	32.018	0.000
	12 months	81.45 ± 3.501		

### 3.2. Anxiety, depression, and subjective wellbeing at different chemotherapy stages

**Figure 1:**
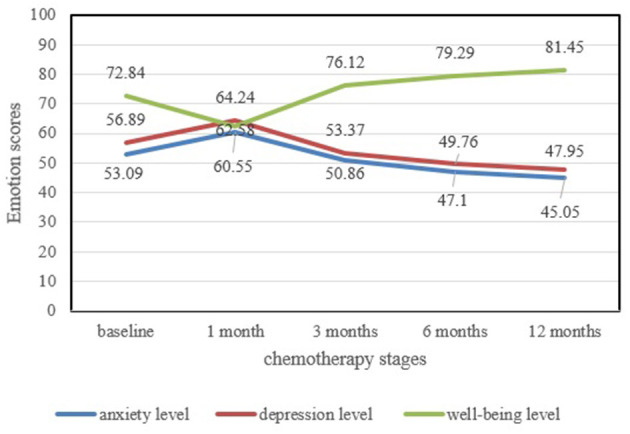
The change in emotion levels and wellbeing levels at different chemotherapy stages.

The changes in the scores of patient's anxiety and depression show a dynamic trend of increasing first and then decreasing with the time of chemotherapy. The scores reach a peak after 1 month of chemotherapy and then become flat after 3 months of chemotherapy gradually, while the changes in the scores of patients' subjective wellbeing show a different trend. They decline first and then rise with the change of chemotherapy time. The scores reach a peak after 1 month of chemotherapy and then become flat out after 3 months of chemotherapy. Figure 1 reveals the development of the patient's anxiety level, depression level, and subjective well-being level.

The statistical results show that there are significant differences in the level of patients' anxiety, depression, and subjective wellbeing at different chemotherapy stages, respectively. All the *P* < 0.05.

### 3.3. The correlation among anxiety, depression, and subjective wellbeing

The Pearson correlation analysis results reveal that there is a negative correlation among the levels of anxiety, depression, and the total level of subjective wellbeing (*r* = −0.605, −0.601), as well as among the level of anxiety, depression, and the sub-scores in each dimension of patients' subjective wellbeing (*r* = −0.431, −0.376; *r* = −0.385, −0.396; *r* = −0.508, −0.518; *r* = −0.484, −0.532; *r* = −0.514, −0.483; *r* = −0.572, −0.576; respectively). Table 2 reveals the correlations in detail.

**Table 2:** Correlation among anxiety, depression, and subjective wellbeing.

**Variable**	**Anxiety**	**Depression**
Subjective wellbeing	−0.605^**^	−0.601^**^
Meet life and interest dimensions	−0.431^**^	−0.376^**^
Dimension of health concerns	−0.385^**^	−0.396^**^
Energy dimension	−0.508^**^	−0.518^**^
Blue and happy mood dimension	−0.484^**^	−0.532^**^
The control dimension of the emotional behavior	−0.514^**^	−0.483^**^
Relax and tension dimensions	−0.572^**^	−0.576^**^

** At level 0.01 (double-tailed), the correlation is significant.

## 4. Discussion

Bladder perfusion chemotherapy is a process in which a doctor infuses chemotherapy drugs into the patient's bladder, aimed to kill the cancer cells remaining in the patient's body and to achieve a therapeutic effect (Liu et al., 2015). As chemotherapy lasts for a long time, patients need to return to the hospital frequently within half a year after surgery. Due to a lack of knowledge of the disease and the fear of adverse reactions, patients are prone to various negative emotions, leading to the loss of confidence in their treatment and the abandonment of chemotherapy (Yu et al., 2019).

Negative emotions such as anxiety and depression are the most common psychological disorders in patients with malignant tumors, which affect the patient's mental health, treatment, and rehabilitation (Nipp et al., 2016). In addition, negative emotions can activate the sympathetic adhesion spot kinase signaling pathway to promote tumor invasion and metastasis, leading to poor prognosis (Cheng et al., 2018). The results of this study show that the average score of patients' anxiety is 53.09 ± 9.370 before chemotherapy and depression is 56.34 ± 11.759, both of which are at the mild level, higher than the results reported by Yang (2016). The results of before chemotherapy in this study are evaluated 1 week after the patients' operation. At that time, the patient's incomplete recovery, bladder pain, decreased self-care ability, and economic burden of surgery led to an increase in their anxiety and depression. Bladder cancer surgery combined with bladder infusion chemotherapy can kill the residual tumor tissue left during the operation and reduce the probability of metastasis of tumor cells that are free in the bladder to a certain extent. However, more than 50% of patients still experience recurrence after surgery, which reduces the patient's confidence in their recovery and increases their negative emotions (Tse et al., 2019). In this study, 30.5% of patients are undergoing reoperation after recurrence, so patients have a high level of anxiety and depression.

Subjective wellbeing is an important indicator for evaluating patients' mental health and quality of life. It can reflect the emotional state of chemotherapy patients for a long period (An et al., 2016). The results of the study show that the average score of patients' subjective wellbeing is 73.59 ± 8.171 before chemotherapy, lower than the national norm (75), indicating that the level of patients' subjective wellbeing is not good. The results of before chemotherapy in the study are evaluated 1 week after the patients' operation. During that period, the high level of negative emotion and psychological burden affected patients' anxiety, depression, and subjective wellbeing.

Negative emotion is a state of uneasiness or unwarranted fear without objective cause. It is the tension about bad situations that will be faced in future, and it will change over time (Yang et al., 2016). When negative emotions occur, the sympathetic nerves regulate the autonomic nervous system, cause changes in endocrine function, and lead to the secretion of multiple catecholamines hormones and thyroid hormones (Rapport et al., 2020). The results of the study show that the patient's anxiety, depression, and subjective wellbeing change dynamically at different stages of chemotherapy. The anxiety and depression levels increase first and then decrease with the chemotherapy, and the subjective wellbeing levels decrease first and then increase. At 1 month of chemotherapy, the patient's anxiety, depression, and subjective wellbeing are in the worst state, and then gradually recover to the level before chemotherapy. After 3 months of chemotherapy, the anxiety, depression, and subjective wellbeing gradually level off. Part of the reason for this phenomenon may be is that the period of the patient's first investigation is only 1 week after their surgery. During that time, they are in a condition with insufficient psychological preparation and fear of chemotherapy, which strengthens the patient's psychological pressure and deepens the negative cognition of the disease and the chemotherapy process (Mei and Sun, 2018). In addition, during the first month of chemotherapy, the artificial ureters are repeatedly inserted into the patient's urethra every week, causing damage to the bladder mucosa, urethral injury, and complications such as frequent urination, urgency, and bladder pain. Complications are most obvious during the first month of chemotherapy, leading to aggravations of anxiety and depression and a decrease in wellbeing in most patients after 3 to 5 weeks of infusion. With the decrease in the frequency of chemotherapy from once a week to once a month, the patient's clinical symptoms are reduced gradually and they accept their disease gradually as well. As a result, their anxiety and depression decrease gradually and their subjective wellbeing improves gradually.

Various psychosocial factors often put patients in a state of chronic stress, leading to negative emotions such as anxiety and depression, which affect the nervous, endocrine, and immune systems (Yin et al., 2019). A previous study has reported that stimulating frontal activity in the brain affects the end product of the hypothalamus–pituitary–adrenal axis in humans, which can regulate emotions (Meng et al., 2019). Another study has proved that maintaining positive emotions in patients with schizophrenia can reduce negative symptoms and improve social functions (Favrod et al., 2019). The results of this study show that patient's anxiety and depression are correlated with their subjective wellbeing negatively, indicating that negative cognitive processing has an inhibitory effect on the patient's subjective wellbeing, while positive cognitive processing can promote the patient's subjective wellbeing (Liu Y. et al., 2020). It can be seen that the level of subjective wellbeing can be improved by reducing the patient's anxiety and depression.

With the improvement of the medical level, the bio-psycho-social medicine model is continuously strengthened. Patients and clinical medical staff pay more and more attention to the patient's mental health and their positive psychological indicators such as subjective wellbeing. However, the measures to directly intervene in subjective wellbeing are complicated, and it is difficult to construct interventions. Therefore, it is possible to improve the patient's subjective wellbeing by interfering with their negative emotions. As the patient's emotional level fluctuates up and down in the first 3 months during chemotherapy, it is better to help patients improve subjective wellbeing by helping them maintain optimistic emotions, increasing their secretion of cortisol, reducing negative emotional experiences, and blocking negative thinking.

## 5. Conclusion

In summary, the changes in the patient's anxiety and depression are most obvious during the first 3 months of chemotherapy, and their subjective wellbeing increases with the decrease in their anxiety and depression. Clinicians can actively take intervention measures in the first 3 months of the patient's chemotherapy, guide patients to relieve their anxiety and depression, help patients improve the quality of their life, and ensure the patients complete their chemotherapy successfully.

## Data availability statement

The original contributions presented in the study are included in the article/supplementary material, further inquiries can be directed to the corresponding author.

## Ethics statement

The studies involving humans were approved by the Committee of Ethics and Integrity in Research with Humans at Tianjin Chengjian University, China. The studies were conducted in accordance with the local legislation and institutional requirements. The participants provided their written informed consent to participate in this study.

## Author contributions

JY, YT, and CY contributed to the conception and design of the study and wrote sections of the manuscript. JY organized the database and wrote the first draft of the manuscript. YT performed the statistical analysis. All authors contributed to the manuscript revision, read, and approved the submitted version.
